# The association between use of snus and quit rates for smoking: results from seven Norwegian cross-sectional studies

**DOI:** 10.1111/j.1360-0443.2010.03122.x

**Published:** 2011-01

**Authors:** Karl E Lund, Janne Scheffels, Ann McNeill

**Affiliations:** 1 Norwegian Institute for Alcohol and Drug Research, University of NottinghamUK; 2 UK Centre for Tobacco Control Studies, Division of Epidemiology & Public Health, University of NottinghamUK

**Keywords:** Former smoking, harm reduction, nicotine, public health, quit rates, smokeless tobacco, smoking cessation, snus

## Abstract

**Aim:**

Swedish studies have shown that experience of using snus is associated with an increased probability of being a former smoker. We examined whether this result is also found in Norway.

**Design:**

Seven cross-sectional data sets collected during the period 2003–08.

**Setting:**

Norway.

**Participants:**

A total of 10 441 ever (current or former) smokers

**Measurements:**

Quit ratios for smoking were compared for people with different histories of snus use. Motive for snus use was examined among combination users (snus and cigarettes). Smoking status was examined among snus users.

**Findings:**

Compared to smokers with no experience of using snus, the quit ratio for smoking was significantly higher for daily snus users in six of seven data sets, significantly higher for former snus users in two of five data sets and significantly lower for occasional snus users in six of seven data sets. Of combination users who used snus daily, 55.3% [confidence interval (CI) 44.7–65.9] reported that their motive for using snus was to quit smoking totally. This motive was reported significantly less often by combination users who used snus occasionally (35.7%, CI 27.3–44.2). Former smokers made up the largest proportion of daily snus users in six of seven data sets. In the remaining data set, that included only the age group 16–20 years, people who had never smoked made up the largest segment of snus users.

**Conclusions:**

Consistent with Swedish studies, Norwegian data shows that experience of using snus is associated with an increased probability of being a former smoker. In Scandinavia, snus may play a role in quitting smoking but other explanations, such as greater motivation to stop in snus users, cannot be ruled out.

## INTRODUCTION

In the European Union, with Sweden as the only exception, snus—a low-nitrosamine smokeless tobacco product—has been banned since 1992. However, with regard to health, there is disagreement about whether the ban is appropriate. The main argument against the ban, raised by bodies such as the Royal College of Physicians of London [1] and the European Respiratory Society [2], is that the ban deprives smokers who are seriously addicted to nicotine a harm-reducing alternative to cigarettes. In the United States, the American Association of Public Health Physicians [3] and a series of eminent tobacco researchers are in favour of including snus within the arsenal of harm-reducing nicotine products. Their recommendations are based upon the conviction that use of snus can contribute to cessation of, or a dramatic reduction in, smoking—the most harmful form of nicotine intake.

However, few randomized controlled trials (RCT) have been carried out to assess the use of snus on smoking cessation. In the absence of experimental studies, observational data have been used to illuminate this issue. In an authoritative report from the European Commission [4], it is claimed that trends in the use of tobacco in Norway indicate that the availability of snus cannot have had much influence on the number of smokers. The reason given is that although snus users are greatly over-represented by men, the rate of reduction in smoking for men is not much greater than that for women. The organization Physicians for a Smoke-free Canada [5] has claimed the same, on the grounds that the reduction in smoking in Norway and Sweden is no greater than in countries such as Canada and Finland, where snus is seldom used.

This type of comparative analysis of trends in the prevalence of smoking for different genders or different countries can, at best, give only a weak indication of the importance of snus for smoking cessation. In epidemiological research of tobacco behaviour, a more precise measure—the quit ratio—can be used to calculate the proportion of people in a population who have stopped smoking [6]. Swedish prospective [7] and retrospective [8] studies have shown that the quit ratio is higher for smokers who use, or have used, snus than for smokers who have never used snus. We have examined whether this same result is also found in a series of recent Norwegian cross-sectional studies, in which it has been possible to measure the quit ratio for daily smoking. We have also studied which motives people who smoke and use snus (combination users) give for their use of snus. The implications of these results for public health are then discussed.

## MATERIALS AND METHODS

The quit ratio for smoking is an expression of the number of former daily smokers as a proportion of the total number of people who have ever smoked daily in a population. It is a statistical measure of smoking cessation activity that is recommended for use in tobacco behaviour research [6]. In order to identify significant differences in quit ratio between different groups of snus users, 95% confidence intervals (CIs) and *P*-values were calculated. As snus use is a predominantly male phenomenon in Norway, no gender-specific results were presented. In the samples with sufficient females for a test, no interaction by gender was observed with regard to quit ratios for smoking across snus status.

The Norwegian Institute for Alcohol and Drug Research collect data on risk-related behaviour in the population on a regular basis. Seven studies in the Institute's databank, carried out since 2003, included questions that made it possible to calculate quit ratios for smoking cigarettes across snus use status (daily, occasional, former and never user). Smoking habits were assessed using the question: ‘Do you smoke?’ (yes, daily/yes, occasionally/no, not at all). In all seven surveys the definition of former smoking was based upon subjective response to a question presented to current non-smokers at the time of the survey: ‘Have you ever smoked daily?’ (yes/no). Current and former snus use was assessed using similar questions.

Study population 1 included 3604 current or former smokers of both genders in the age group 16–74 years. The subjects were identified from a data pool from yearly representative surveys of tobacco behaviour carried out by Statistics Norway (SSB) by telephone for the years 2003–08, and included 7500 respondents in total. The mean response rate for the period was 67%. The material and methods for these surveys have been described previously [9]. The motives for using snus among dual users of snus and cigarettes in this study were assessed by asking how well—on a scale from 1 (apply fully) to 5 (do not apply at all)—the statements listed in Table 4 described their situation. This question was included in the surveys for the period 2005–08. The percentages for those who scored 1 or 2 on the scales are displayed in Table 4. In order to identify significant differences in motives for snus use between daily and occasional snus users, 95% CIs were calculated.

**Table 4:** Survey 1: Percentage (95% CI) of combination users of snus and cigarettes agreeing with statements concerning motives for snus use.

	Daily smokers who use snus daily (n = 85)	Daily smokers who use snus occasionally (n = 123)	All smokers and snus users (n = 208)
Use snus to quit smoking completely	55.3 (44.7–65.9)	35.7 (27.3–44.2)	43.8 (37.1–50.5)
Use snus to reduce the amount of cigarettes they smoke	64.7 (54.5–74.9)	50.4 (41.6–59.2)	56.3 (49.6–63.0)
Use snus to replace cigarettes in places where smoking is not allowed	65.9 (55.8–76.0)	54.4 (45.6–63.2)	59.1 (52.4–65.8)

Study population 2 included 423 current or former smokers of both genders in the age group 16–20 years. This sample was drawn from a national representative survey of tobacco behaviour carried out by telephone by Synovate, Norway in September 2007 among 2415 people. The material and methods have been described previously [10].

Study population 3 included 790 women and men born later than 1970 who were students at Oslo University (UiO) in 2007, and who reported that previously or at the time of the survey they had smoked cigarettes daily. They were identified from a survey of 1655 students with information about use of alcohol, drugs and tobacco. The survey was carried out by a mailed questionnaire by the Foundation for Student Life in Oslo (SIO), in cooperation with the Norwegian Institute for Alcohol and Drug Research (SIRUS). The response rate was 57%. The material and methods have been described previously [11].

Study population 4 included 2018 smokers and former smokers of both genders in the age group 15–91 years in 2007. These people were selected from Synovate, Norway's national representative Norwegian Monitor Survey of 3683 people carried out by home visits. The material and methods have been described previously [12].

Study population 5 included 729 smokers and former smokers of both genders in the age group 21–30 years, selected from a national survey of 2362 people carried out by mailed questionnaire by SIRUS in 2006. The response rate was 42%. The material and methods have been described previously [13].

Study population 6 included 639 smokers and former smokers of both genders in the age group 21–30 years selected from a survey of 2270 young adults in the Norwegian capitol Oslo, carried out by mailed questionnaire by SIRUS in 2006. The response rate was 45%. The material and methods have been described previously [13].

Study population 7 consisted of 2572 male ever smokers aged 20–50 years selected randomly from a national representative web panel. Of those invited to participate, 7170 men (48.6%) responded to an electronic questionnaire. The material and methods have been described previously [14].

## RESULTS

Table 1 presents information about the seven surveys with 27 955 respondents in total, in which questions about use of tobacco and snus were asked. A total of 10 441 people (both users and non-users of snus) reported that they had either smoked daily previously but had quit (5144) or that they smoked daily at the time of the survey (5207) (Table 2). In the student population in Oslo (study 3), the quit ratio for smoking was 67.4% (95% CI 63.1–71.7). This was significantly higher than in the other samples. The lowest quit ratio of 32.2% (95% CI 27.8–36.7) was observed in the nationally representative sample of young people in the age group 16–20 years (study 2). The quit ratio was 52% in both of the two national representative samples that had the same age and gender distribution as the normal population (Table 2).

**Table 1:** Portfolio of studies.

Survey	Data collector	Survey carried out on behalf of:	Year	Characteristics of the sample	Size of the sample	Detailed description of the data
1	SSB	Norwegian Directorate of Health	2003–08	Nationally representative women/men 16–74 years	7500	Nadim (2007)
2	Synnovate	Norwegian Directorate of Health	2007	National women/men 16–20 years	2415	Øverland *et al*. (2008)
3	SIRUS	SIRUS/SIO	2006	Students at University of Oslo born after 1970	1655	Lund *et al*. (2008)
4	Norsk Monitor	Synnovate	2007	Nationally representative women/men 15–91 years	3683	Hellevik (2008)
5	SIRUS	SIRUS	2006	National women/men 21–30 years	2362	Lund *et al*. (2007)
6	SIRUS	SIRUS	2006	Oslo women/men 21–30 years	2270	Lund *et al*. (2007)
7	SIRUS	Norwegian Directorate of Health	2007	National men 20–50 years	7170	Lund *et al*. (2010)

SIRUS: Norwegian Institute for Alcohol and Drug Research; SIO: Foundation for Student Life in Oslo; SSB: Statistics Norway.

**Table 2:** Number of former and current daily smokers, and quit ratio for daily cigarette smoking in seven Norwegian studies.

Study	Former daily smokers	Current daily smokers	Total	Quit ratio (95% CI)
1	1870	1734	3604	51.9 (50.3–53.5)
2	136	287	423	32.2 (27.8–36.7)
3	296	162	458	67.4 (63.1–71.7)
4	1041	975	2016	51.6 (49.4–53.8)
5	318	411	729	43.6 (40.0–47.2)
6	328	311	639	51.3 (47.4–55.2)
7	1155	1417	2572	44.9 (43.0–46.8)
Total	5144	5297	10441	

CI: confidence interval.

In six of the seven studies the quit ratio for smoking was significantly higher for daily snus users than for people with no experience of using snus (Table 3). The same finding was observed for people who at the time of the study reported that they had used snus previously in five of the six studies, but the differences were significant in only two of the studies. The quit ratio for smoking for people who used snus occasionally was significantly lower than for people who had no experience of snus use in six of seven studies.

**Table 3:** Quit ratio (95% Confidence interval) for daily cigarette smoking according to snus use in seven Norwegian surveys. *P*-values contrasts ‘never used’.

	Current status of snus use						
	
Survey	Daily user	P	Occasional user	P	Former user	P	Never used
1	80.4 (74.9–85.9)	<0.001	32.0 (25.6–38.4)	<0.001	45.5 (39.9–51.1)	0.033	51.9 (50.1–53.7)
2	54.8 (45.2–64.9)	<0.001	21.7 (4.9–38.6)	0.895	31.1 (20.6–41.7)	0.178	22.9 (17.4–28.4)
3	81.1 (68.5–93.7)	0.010	42.7 (32.0–53.4)	0.001	85.7 (75.9–95.5)	<0.001	62.7 (56.9–68.4)
4	61.6 (53.5–69.7)	0.035	27.6 (19.7–35.5)	<0.001	–	–	52.5 (50.2–54.8)
5	75.4 (65.2–85.6)	<0.001	21.2 (14.8–27.6)	<0.001	53.9 (42.7–65.1)	0.015	44.9 (40.2–49.6)
6	89.7 (81.9–97.5)	<0.001	24.4 (17.1–31.8)	<0.001	83.9 (74.3–93.5)	<0.001	50.0 (45.1–54.9)
7	72.7 (68.2–77.2)	<0.001	17.4 (13.6–21.2)	<0.001	48.8 (44.4–53.2)	0.037	43.3 (40.6–46.0)

For snus users who also smoked cigarettes on a daily basis, 43.8% of them reported that they used snus to quit smoking, 56.3% to reduce their smoking and 59.1% to replace cigarettes in places where smoking is not allowed (Table 4). All three motives for using snus were more common for smokers who used snus on a daily basis compared to smokers who used snus occasionally, but this was significant only for those reporting using snus to quit smoking: 55.3% daily users (95% CI 44.7–65.9) compared with 35.7% occasional users (95% CI 27.3–44.2).

In the survey that included only young people in the age group 16–20 years (study 2), 42.8% of daily snus users were without previous smoking experience, while only 17.9% were former smokers (Table 5). In the other six surveys the proportion of snus users who were former smokers formed the largest group among snus users (varying between 34.4 and 42.5% of all snus users). The proportion of snus users who had never smoked cigarettes varied from 18.8 to 31% in the same six surveys. A small minority of snus users (3.1–10.6%) smoked daily, but many more smoked occasionally (16–35%).

**Table 5:** Smoking status of daily snus users. Percent. Results from seven Norwegian surveys.

Survey	Daily smoker (%)	Occasional smoker (%)	Former smoker (%)	Never smoked (%)	n
1	10.6	22.2	39.6	27.6	369
2	14.8	24.5	17.9	42.8	318
3	8.8	35.0	38.5	18.8	80
4	10.5	16.0	42.5	31.0	200
5	7.9	27.0	41.4	23.7	215
6	3.1	27.6	41.8	27.6	196
7	9.4	32.1	34.4	24.1	1113

## DISCUSSION

Snus appeared to play a role in quitting smoking: daily snus use and, to a lesser extent, former snus use, were associated with being a former smoker across most of the included studies; occasional snus use was less likely to be associated with being a former smoker. However, the results must be interpreted with caution, as with non-randomized observational studies we cannot exclude the danger of selection bias in the groups that are compared.

### Low quit ratio for smoking among people with a low use of snus

In contrast to Sweden, where nearly all snus users are daily users, almost half of Norwegian snus users use snus only occasionally [15]. In this group, the quit ratio for smoking was low (Table 3), and the proportion of combination users of snus and cigarettes was accordingly high. A possible interpretation is that occasional snus users have a stable combination use, in which snus is used more as a substitute for cigarettes, for example in the steadily increasing social arenas where cigarette smoking is undesirable. An alternative interpretation of this relationship is that many of these low–frequent snus users at the time of the interview were in an incomplete transition phase of stopping smoking daily, and that they will replace cigarettes with daily use of snus later. A clarification of the two hypotheses requires longitudinal data.

### The consequences for public health

Use of snus can damage health in a number of different ways, some serious and some less serious, but systematic literature reviews [4] and register-based prospective studies [16] have concluded that snus is much less hazardous than smoking. It is estimated that the reduction in risk by switching from cigarettes to snus is at least 90% [17]. If snus increases the rate of smoking cessation, what are the consequences for public health if, overall, the use of snus increases? The extent and nature of the impact on public health will depend upon the relative risk hazard of snus and smoking, and the relative uptake and use by smokers and non-smokers.

To identify the net effect of snus use from a public health perspective is a complicated task. However, the conditions for carrying out this task are best in countries such as Norway and Sweden, using our observational data on the transition between cigarettes and snus.

In all the studies described here, except the one of people under 20 years of age, people who had quit smoking formed the largest group of snus users. The proportion of snus users who had no previous experience of smoking was less than the proportion of ex-smokers in all the studies, except for the youngest respondents. One frequently reported study [18] found that 14–25 non-smokers would have to begin to use snus in order to cancel out the positive health effects from each smoker who transferred from cigarettes to snus. The results from the studies presented here suggest that in Norway, as in Sweden, snus users are more likely to be recruited from smokers than from non-smokers. This supports the hypothesis that availability of snus has more positive effects on public health than negative effects—at least in these two countries.

A small minority of snus users (3.1–10.6%) smoked daily, while many more smoked occasionally (16–35%). As we lack data about reduced smoking frequency among these combination users—as shown in Sweden [19]—it is difficult to draw any conclusions about whether this combination use is more or less damaging than the amount of smoking that would have taken place without snus use.

### Smoking among young snus users

The picture was different in the survey that included only young people in the age group 16–20 years (survey 2). In this survey as many as 42.8% of snus users had no smoking experience, while only 17.9% were former smokers. This result probably emanates from the fact that the general quit ratio for smoking is very low in such a young age group (Table 2). Thus snus, probably along with medicinal products, is not used as a method for quitting smoking to the same degree among young people as among older established smokers.

The largest increase in use of snus during the last few years has been among young people [15]. If almost half these recruits are primary users, as our surveys indicate, there is cause for concern that increased use of snus does not result in harm reduction in this cohort, but increased health risk. Conversely, we might assume that a segment of primary users are young people who would otherwise have begun to smoke if snus had not been available and would therefore have been exposed to a product with greater health risk. Studies have shown that young people who initiate tobacco use through using snus have many of the same predisposing factors as young smokers [20], and that this may indicate that the products recruit users from the same segment of the population. Indeed, a longitudinal study from Sweden [21] has shown that use of snus reduces the risk of starting to smoke among young people, when known predictors for starting to smoke are controlled for. Data from the United States are more inconsistent [22,23]. At any rate, a large proportion of people must be recruited to be primary users of snus in order to cancel the health benefits for every young person who starts to use snus instead of smoking [18,24].

### The problem of measuring quitting activity

Even assuming that the comparison of the quit ratio for smoking by use of snus gives a much more valid indication of the importance of snus for quitting smoking than gender-specific or country-specific comparisons of the trend in the prevalence of smoking, the quit ratio is still a fairly crude measure of quitting activity [6]. Although former smoking was defined consistently across these seven studies, our definition did not distinguish between former smokers according to how long ago they had stopped. This is a weakness.

## CONCLUSION

In Norway, as in Sweden, the quit ratio for daily smoking is higher for daily snus users than for people who have never used snus. People who use snus occasionally have the lowest quit ratio for smoking. Former smokers make up the largest segment of Norwegian snus users, except among the 16–20-year-olds. These results indicate that for many smokers use of snus may represent an exit from cigarette smoking. The larger number of primary snus users in the younger age group may lead to a change in the future composition of snus users according to their previous smoking experience.

### Declarations of interest

None.

## References

[1] Royal College of Physicians (2007). Harm Reduction in Nicotine Addiction. Helping People Who Can't Quit.

[2] Martinet Y, Fagerström K, McNeill A, Godfrey F (2006). Harm reduction strategies for tobacco smoking: report from an ERS Research Seminar. Sleep Breath.

[3] American Association of Public Health Physicians (2008). The Case for Harm Reduction for Control of Tobacco-Related Illness and Death.

[4] Scientific Committee on Emerging and Newly Identified Health Risks (SCENIHR) (2008). Health Effects of Smokeless Tobacco Products.

[5] Physicians for a Smoke-free Canada (2007). The Snus Experience.

[6] International Agency for Research on Cancer/World Health Organization (IARC/WHO) (2008). Methods for Evaluating Tobacco Control Policies.

[7] Stenbeck M, Hagquist C, Rosén M (2009). The association of snus and smoking behavior: a cohort analysis of Swedish males in the 1990s. Addiction.

[8] Ramstrom L, Foulds J (2006). Role of snus in initiation and cessation of tobacco smoking in Sweden. Tob Control.

[9] Nadim M (2007). Reise- Og Ferieundersøkelsen 2006 [Travel- and holiday survey 2006].

[10] Øverland S, Hetland J, Aarø LE (2008). Relative harm of snus and cigarettes: what do Norwegian adolescents say?. Tob Control.

[11] Lund KE, Tefre E, Amundsen A, Nordlund S (2008). Røyking, Bruk Av Snus Og Annen Risikoatferd Blant Studenter [Smoking, use of snus and other risk behaviour among university students]. Tidskr nor legeforen.

[12] Hellevik O (2008). Jakten på den norske lykken [On the lookout for the Norwegian happiness].

[13] Lund MKØ, Skretting A, Lund KE (2007). Rusmiddelbruk blant unge voksne, 21–30 år Resultater fra spørreskjemaundersøkelser 1998, 2002 og 2006 [Use of drugs among young adults aged 21–30 years. Results from the surveys 1998, 2002 and 2006].

[14] Lund KE, McNeill A, Scheffels J (2010). The use of snus for quitting smoking compared with medicinal products. Nicotine Tob Res.

[15] Lund M, Lindbak R (2007). Norwegian Tobacco Statistics 1973–2006.

[16] Henley SJ, Connell CJ, Richter P, Husten C, Pechacek T, Calle E (2007). Tobacco-related disease mortality among men who switched from cigarettes to spit tobacco. Tob Control.

[17] Levy DT, Mumford EA, Cummings KM, Gilpin EA, Giovino G, Hyland A, Sweanor D (2004). The relative risks of a low-nitrosamine smokeless tobacco product compared with smoking cigarettes: estimates of a panel of experts. Cancer Epidemiol Biomakers Prev.

[18] Gartner CE, Hall WH, Vos TH, Bertram MY, Wallace AL, Lim SS (2007). Assessment of Swedish snus for tobacco harm reduction: an epidemiological modeling study. Lancet.

[19] Gilljam H, Galanti MR (2003). Role of snus (oral moist snuff) in smoking cessation and smoking reduction in Sweden. Addiction.

[20] Hagquist CEI (2006). Health inequalities among adolescents: the impact of academic orientation and parents' education. Eur J Public Health.

[21] Galanti MR, Rosendahl I, Wickholm S (2008). The development of tobacco use in adolescence among ‘snus starters’ and ‘cigarette starters’: an analysis of the Swedish ‘BROMS’ cohort. Nicotine Tob Res.

[22] Timberlake DS, Huh J, Lakon CM (2009). Use of propensity score matching in evaluating smokeless tobacco as a gateway to smoking. Nicotine Tob Res.

[23] Severson HH, Forester KA, Biglan A (2007). Use of smokeless tobacco is a risk factor for cigarette smoking. Nicotine Tob Res.

[24] Kozlowski LT, Strasser AA, Giovino GA, Erickson PA, Terza JV (2001). Applying the risk/use equilibrium: use medicinal nicotine now for harm reduction. Tob Control.

